# The Pre-Interictal Network State in Idiopathic Generalized Epilepsies

**DOI:** 10.3390/brainsci13121671

**Published:** 2023-12-02

**Authors:** Dimitrios Pitetzis, Christos Frantzidis, Elizabeth Psoma, Smaranda Nafsika Ketseridou, Georgia Deretzi, Anna Kalogera-Fountzila, Panagiotis D. Bamidis, Martha Spilioti

**Affiliations:** 1 Department of Neurology, Papageorgiou General Hospital, 56403 Thessaloniki, Greece; gderetzi@gmail.com; 2 Lab of Medical Physics and Digital Innovation, School of Medicine, Faculty of Health Sciences, Aristotle University of Thessaloniki, 54124 Thessaloniki, Greece; christosfrantzidis@gmail.com (C.F.); smarantan@auth.gr (S.N.K.); pdbamidis@gmail.com (P.D.B.); 3 School of Computer Science, University of Lincoln, Lincoln LN6 7TS, UK; 4 Department of Radiology, AHEPA General Hospital, School of Medicine, Faculty of Health Sciences, Aristotle University of Thessaloniki, 54636 Thessaloniki, Greece; elizabethpsoma@gmail.com (E.P.); ankaloge@icloud.com (A.K.-F.); 5 1st Department of Neurology, AHEPA General Hospital, School of Medicine, Faculty of Health Sciences, Aristotle University of Thessaloniki, 54636 Thessaloniki, Greece; marthags@auth.gr

**Keywords:** EEG, cortical connectivity, graph theory analysis

## Abstract

Generalized spike wave discharges (GSWDs) are the typical electroencephalographic findings of Idiopathic Generalized Epilepsies (IGEs). These discharges are either interictal or ictal and recent evidence suggests differences in their pathogenesis. The aim of this study is to investigate, through functional connectivity analysis, the pre-interictal network state in IGEs, which precedes the formation of the interictal GSWDs. A high-density electroencephalogram (HD-EEG) was recorded in twenty-one patients with IGEs, and cortical connectivity was analyzed based on lagged coherence and individual anatomy. Graph theory analysis was used to estimate network features, assessed using the characteristic path length and clustering coefficient. The functional connectivity analysis identified two distinct networks during the pre-interictal state. These networks exhibited reversed connectivity attributes, reflecting synchronized activity at 3–4 Hz (delta2), and desynchronized activity at 8–10.5 Hz (alpha1). The delta2 network exhibited a statistically significant (*p* < 0.001) decrease in characteristic path length and an increase in the mean clustering coefficient. In contrast, the alpha1 network showed opposite trends in these features. The nodes influencing this state were primarily localized in the default mode network (DMN), dorsal attention network (DAN), visual network (VIS), and thalami. In conclusion, the coupling of two networks defined the pre-interictal state in IGEs. This state might be considered as a favorable condition for the generation of interictal GSWDs.

## 1. Introduction

The network connectivity and graph theory provide the necessary tools to investigate the intricate nature of the human brain connectome. The graph theory defines a brain network as a set of nodes (anatomical regions or electrodes) and interconnecting edges (structural or functional connections) [1]. The functional connectivity between different brain regions unveils both their interactions and their network topology, even in the absence of direct anatomical connections [2]. The graph theory analysis provides data to identify the most influential nodes within networks [3]. This information could be utilized to study the connectivity states of brain networks [4]. The progress in the field of neuroscience has led to the definition of epilepsy as a network disorder. Epileptic networks are affected by complex inhibitory and excitatory dynamics. If unbalanced, these dynamics can cause changes in the synchronization between brain regions, resulting in seizures [5].

Idiopathic Generalized Epilepsies (IGEs) encompass a spectrum of clinically overlapping epileptic syndromes that are part of the genetic generalized epilepsies. These syndromes include Generalized Tonic-Clonic Seizures alone (GTCSa), Juvenile Myoclonic Epilepsy (JME), Juvenile Absence Epilepsy (JAE), and Childhood absence epilepsy (CAE) [6]. The main characteristics of the electroencephalogram (EEG) in IGEs are normal background and generalized spike–wave discharges (GSWDs), typically with a frequency ranging from 2.5 to 5.5 Hz. Furthermore, atypical EEG findings are also notable and include asymmetry, focalities, generalized paroxysmal fast activity, and aberrant morphology of spike–wave complexes, such as waves without spikes [7,8].

In IGEs, the occurrence of GSWDs can be interpreted as a manifestation of a network disorder during ictal (seizure), as well as interictal activity (interval between seizures). Various theories have been proposed on epileptogenesis in IGES. The cortico-reticular theory proposes that GSWDs are a pathological expression of the thalamocortical pathway. On the other hand, the cortical focus theory assumes that cortical areas are active before the generation of the GSWDs, while an alternating driving of the thalamus and the cortex sustains the epileptic activity [9].

Another crucial aspect of the epileptogenesis in IGEs refers to the preictal brain state, during which brain activity inevitably progresses towards the generation of GSWDs. EEG functional connectivity studies on the preictal activity in IGEs have revealed variations in network topology, preceding the onset of epileptic activity [10,11].

The interictal GSWDs have similar electroencephalographic morphological features with the ictal ones, although they are not accompanied by seizures or consciousness impairment. A magnetoencephalography (MEG) study on absence epilepsy highlighted that the interictal discharges were associated with decreased connectivity in the central executive network, default mode network (DMN), and attention network. The preservation of consciousness during interictal GSWDs was attributed to the activation of the posterior cingulate cortex and precuneus in the theta and alpha frequency bands. These connectivity alterations during the interictal GSWDs impeded hyperexcitability, which is crucial for epileptogenesis [12]; similar alterations have also been observed in focal epilepsies [13,14].

The pathophysiological mechanisms of ictal, interictal, and preictal states in IGEs have been well studied. There are limited heterogeneous functional connectivity data concerning the state preceding the interictal GSWDs (pre-interictal) in IGEs. In JME, one study showed a coupling of opposite dynamic states, in the gamma and beta frequency bands, within the frontoparietal regions [15], whereas another one displayed an increased connectivity in the delta frequency band between the cingulate gyrus and cuneus [16]. The aim of this study is to investigate the pre-interictal functional connectivity state in IGEs. We evaluate the following hypotheses: (i) there are network alterations between the resting and the pre-interictal state, and (ii) there are nodes with a distinct influence on the pre-interictal state. We examine these hypotheses by analyzing the cortical connectivity based on data obtained with a high-density EEG (HD-EEG) and brain magnetic resonance imaging (MRI 1.5 T).

## 2. Materials and Methods

### 2.1. Patient Characteristics

The individuals included in the study were selected from the outpatient clinic of the First Department of Neurology at the AHEPA Hospital. To be included in the study, individuals needed to fulfill the following criteria: (a) have a confirmed diagnosis of IGE according to the ILAE definition [17], (b) be above 18 years of age, and (c) have normal brain MRI results according to conventional diagnostic techniques. Prior to their participation, patients were given comprehensive information about the study and were required to complete a signed consent form. The bioethics committee of the Aristotle University of Thessaloniki approved this research under protocol number 1.74. The sample size for the study consisted of twenty-one patients diagnosed with IGE. The clinical and EEG characteristics of the patients are summarized in Table 1.

### 2.2. MRI Acquisition

A brain MRI scan was conducted for all patients at Siemens 1.5 T in AHEPA University Hospital using an epilepsy protocol. The MRI sequences included 3D T1WI, T2w, FLAIR, susceptibility-weighted imaging (SWI), or T2-star (T2*) WI, as well as diffusion-weighted imaging (DWI) and apparent diffusion coefficient (ADC) mapping. The 3D T1 sequence was acquired using the following scanning parameters: slice thickness 1.6 mm, repetition time 25 ms, echo time 4 ms. The latter sequence was utilized for the head modelling process.

### 2.3. EEG Recording

The HD-EEG recordings were conducted by a professionally trained technician using a Nihon-Kohden EEG device with 128 electrodes placed on the scalp. Four specific points, namely the nasion, inion, left pre-auricular, and right pre-auricular, were used as landmarks. The recordings had a sampling rate of 1000 Hz. Electrocardiographic activity and electrooculograms were captured using bipolar electrodes. Two reference electrodes were attached to the mastoids, and a ground electrode was also used. Each recording had a duration of 30 min. The recordings were conducted in a specially designed room that provided electrical shielding and sound and light isolation, as well as dim lighting. The patients were comfortably seated in an armchair during the recordings.

### 2.4. EEG Analysis

We utilized the Brainstorm freeware [18] for EEG data processing, source localization, and cortical connectivity analysis, as described in the following steps. The EEG captures both cerebral activity and electrical disturbances called artifacts. To eliminate the spectral content not relevant to brain activity, such as power line noise, the following third-order Butterworth filters were utilized:

High-pass filter/cut-off frequency at 0.5 Hz;

Low-pass filter/cut-off frequency at 50 Hz;

Band-stop filter/ranging from 47 to 53 Hz;

Band-stop filter/ranging from 97 to 103 Hz;

Common average re-referencing [19,20].

Epochs were, then, preprocessed with Independent Component Analysis (ICA) to remove artifact sources, including the heartbeats, ocular movements, and muscle activity [21].

### 2.5. Epoch Selection

For each subject, a neurophysiology specialist chose two sets of epochs of 6 s each.

1. Epochs of brain activity prior to the first interictal epileptiform discharge. We named these epochs as pre-interictal.
2. Resting-state (RS) epochs with a difference of at least three minutes from the GSWDs.

For every individual, the count of resting-state (RS) epochs was adjusted to match the count of pre-interictal epochs.

### 2.6. Source Localization

Source localization involves resolving both the forward and inverse problems. The former pertains to estimating a model that clarifies the measurement of brain activity from sensor electrodes on the scalp. Addressing the forward problem entails determining an electrical source configuration, encompassing activated brain neurons, sensor electrode coordinates, and electrode alignment on the head model. Meanwhile, the inverse problem seeks to identify sources responsible for the recorded potential at the sensor level. Numerous factors, such as errors in head modeling and EEG noise (external or biological), could impact the precision with which a source is identified [22,23].

The cortex envelope of the individual brain anatomy, obtained from the T13D MRI sequence, was extracted using the CAT12 toolbox [24]. Subsequently, a linear MNI normalization and the coordinates of the anatomical fiducials (nasion and pre-auricular points), were computed with the Brainstorm software (version 3.230427). Each cortex surface was downsampled to 15,000 vertices, while the Open MEEG Boundary Elements Method was utilized for head modeling [25]. For the computation of the inverse problem, the constrained standardized low-resolution brain electromagnetic tomography (sLORETA) methodology was employed. The noise covariance statistics were computed using the recorded data, and only the diagonal elements were employed [26]. 

### 2.7. Regions of Interest

To define regions of interest, we first averaged the individual source maps of each condition at the patient level. Then, the rectified averages were projected to the ICBM 152 Brain Anatomy Template. Subsequently, the group-level average was calculated for each condition, followed by the difference between the averages. We applied an arbitrary threshold of 10% to the difference values. Voxels surpassing this threshold were defined as the regions of interest (ROIs). The ROIs were parcellated into anatomical areas based on the Desikan–Killiany atlas [27], including the surface of the thalami. The scouts, designed on the anatomy template, were then projected to the individual anatomy of each patient. The average of the signals of the above suprathreshold voxel of the ROIs generated the representative scout series.

### 2.8. Cortical Functional Connectivity

Connectivity in the context of EEG refers to the investigation of interactions between two or more EEG signals. The computation of EEG connectivity involves analyzing activity potentials acquired from the scalp. Functional connectivity specifically addresses statistical correlations among activities in different brain regions, assessable at either the sensor or cortical level [4].

We estimated source-level connectivity using the lagged coherence method. This technique examines the delayed linear relationship between sets of multivariate time series. Lagged coherence utilizes an orthogonalization technique that yields zero values exclusively in the presence of linear mixing between signals (crosstalk), while also possessing a magnitude unaffected by the extent of crosstalk. Consequently, it relies solely on the genuine interactions between the two signals [28]. It is resistant to immediate volume conduction effects and low spatial resolution [29]. 

Lagged coherence serves as a reliable measure for assessing brain interactions derived from EEG/MEG data [30,31]. This estimator has been previously utilized in other studies of cortical connectivity [32,33]. The zero-lag-removed general coherence is calculated with the following formula:

(1) ρGL=Im⁡Syxωsyyωsxxω-Re⁡syxω2

where ρ_GL_ is the lagged coherence, yω and xω are the Fourier transforms of yjt and xjt time series, respectively, lm(S_yxω_) and Re(S_yxω_) are the imaginary and real parts, respectively, and s_yyω_ and s_xxω_ are the pure real variances [34]. Lagged coherence was estimated for the following frequency bands: delta1 = 0.5–3, delta2 = 3–4, theta1 = 4–5.5, theta2 = 5.5–7, alpha1 = 8–10.5, alpha2 = 10.5–13, and beta = 14 to 30 Hz.

### 2.9. Network Analysis

We processed the weighted undirected matrices, which include nodes representing ROIs and their weights derived from lagged coherence calculation, using Network-Based Statistics [35]. To identify networks exhibiting statistically significant altered connectivity between the two states, the connectivity matrices were compared across different frequency bands. For the statistical model, the following options were chosen: the design matrix included one group of subjects and two conditions, the statistical test utilized a t-test, and the threshold value was set at 1.5 (T = 1.5). A total of five thousand permutations were performed, and both contrasts were evaluated. The output yielded a binary adjacency matrix network, where a value of “1” indicated a statistically significant probability of connectivity between two nodes, whereas a value of “0” indicated the opposite in the adjacency matrix.

### 2.10. Graph Theory Analysis

Graph theory was employed to analyze the topology of the statistically significant networks utilizing weights derived from lagged coherence values. Network functions related to the integration and segregation of information were investigated using the characteristic path length and the weighted clustering coefficient, respectively.

The characteristic path length is a metric that describes the average shortest path length between all pairs of nodes in a network. It is associated with the network efficiency of information transfer between nodes [36]. The weighted characteristic path length is calculated using the following mathematical formula:

(2) $$L^{w}=\frac{1}{n}\sum_{i\in N}\frac{\sum_{j\in N,\;j\neq i}d_{\mathrm{ij}}^{w}}{n-1}$$

where N is the set of all nodes, n is the number of nodes, links (i, j) are associated with connection weights w_ij_, and $d_{\mathrm{ij}}^{w}$ is the shortest weighted path length between i and j [37].

The mean clustering coefficient (mean C) measures the propensity of a network to establish local circuits around individual nodes, reflecting its capacity for information processing [36]. The weighted clustering coefficient is determined using the following mathematical formula:

(3) $$C^{w}=\frac{1}{n}\sum_{i\in N}\frac{2t_{i}^{w}}{k_{i}(k_{j}-1)}$$

where N is the set of all nodes, n is the number of nodes, links (i, j) are associated with connection weights w_ij_, k_i_ is the degree of a node i, and $t_{i}^{w}$ is the weighted geometric mean of triangles around i [37].

To determine the role of each node, we computed the spreading and hubness score. This step was performed using the RStudio software (version 4.2.3) [38] and the “influential” package in R. The spreading score reflects the node’s ability to propagate information and is calculated using the following formula:

Spreading score = (NC′i + CR′i) × (BC′i + CI′i)(4)

Here, NC′i, CR′i, BC′i, and CI′i represent the normalized neighborhood connectivity, ClusterRank, betweenness centrality, and collective influence of node i, respectively. Nodes exhibiting statistically significant alterations in their spreading scores were considered as spreader nodes.

On the other hand, the hubness score measures the impact of each node within its domain. It is computed using the following formula:

Hubness score = DC′i + LHindexi(5)

In this equation, DC′i and LHindexi represent the normalized degree centrality and local H index of node i, respectively [3]. Nodes with a hubness score higher than the mean value plus one standard deviation were arbitrarily defined as hubs in the case of normal distributions of hubness values. Conversely, the threshold was set as the median value plus the interquartile range.

### 2.11. Statistical Analysis

The statistical analysis was conducted using IBM SPSS Statistics software version 20. Initially, the normality assumption of the differences in characteristic path length and mean clustering coefficient values was assessed across both states and all frequencies. The Shapiro–Wilk test was used for this purpose, taking into consideration the sample size. If the variable did not follow a normal distribution, the Wilcoxon signed-rank test was utilized. The Bonferroni correction method was applied to identify statistically significant differences.

For the analysis of the spreading score, a permutation test was conducted with five thousand permutations. The False Discovery Rate (FDR) correction method was employed to account for multiple comparisons.

### 2.12. Network Visualization

The statistically significant networks were visualized utilizing the BrainNet Viewer toolbox (version 1.7) [39] based on the ICMB152 template and the centroid coordinates of the ROIs, both provided by the toolbox. 

### 2.13. Methodology Flowchart

**Figure 1:**
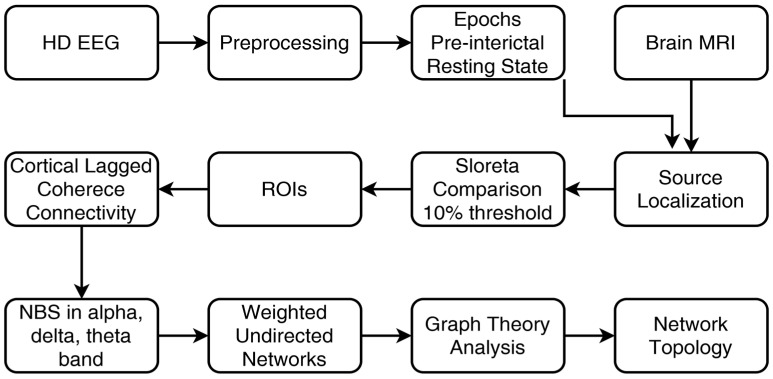
Methodology flowchart.

Figure 1 illustrates the integration of data from the HD-EEG and brain MRI in implementing source and connectivity analysis.

## 3. Results

In this study, an HD-EEG was recorded in twenty-one patients with IGEs, and individual anatomy was obtained with brain MRI 1.5 T. Twenty-seven pre-interictal epochs were recorded. Cortical connectivity analysis was conducted through the estimation of lagged coherence. Network properties were assessed through the characteristic path length and mean clustering coefficient. Nodal properties were investigated via the hubness and spreading score. The details of the results are described below.

### 3.1. Regions of Interest

**Figure 2:**
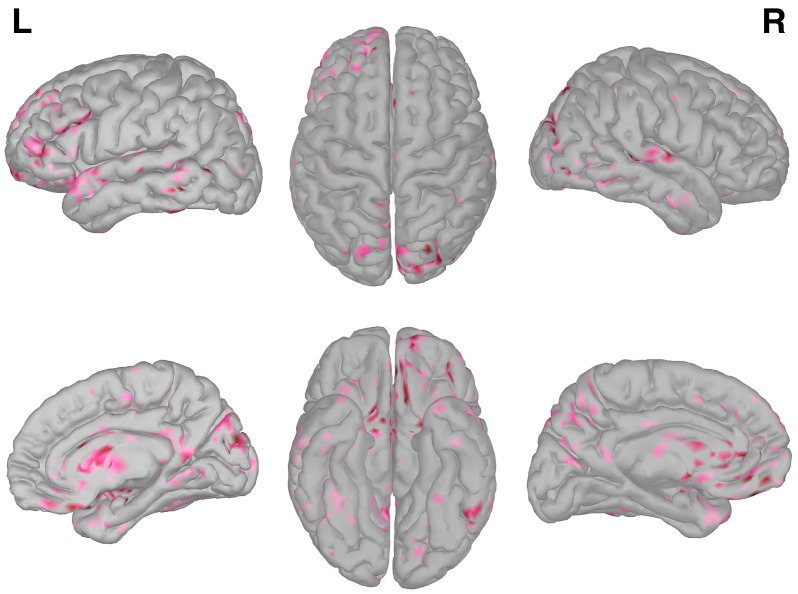
ROI map consisting of suprathreshold vertices. Darker color indicates higher difference between the pre-interictal and resting-state sLORETA values. L: left, R: right.

The arbitrary threshold of 10% to the grand average difference of pre-interictal and resting-state sLORETA values distinguished 812 suprathreshold vertices of the ICBM 152 template anatomy. Areas without significant differences in sLORETA values included the transverse temporal gyri, supramarginal gyri, left postcentral gyrus, parahippocampal gyri, left paracentral lobule, left fusiform gyrus, right frontal pole, left caudal middle frontal gyrus, right caudal anterior cingulate gyrus, and the banks of the left superior temporal sulcus. The suprathreshold vertices prior the anatomic parcellation according to the Desikan–Killiany atlas are visualized in Figure 2.

### 3.2. Network Characteristics

In this HD-EEG cortical lagged coherence analysis, we observed two networks with distinct topologies during the pre-interictal state. The first network (delta2) had increased connectivity at 3–4 Hz and presented a decreased characteristic path length, as well as an increased mean clustering coefficient. The second network (alpha1) was characterized by opposed dynamics to the first one, with decreased connectivity at 8–10.5 Hz, an increased characteristic path length, and a decreased mean clustering coefficient. No statistically significantly altered network was identified with the NBS method in delta1, theta1, theta2, alpha2, and beta bands. 

**Figure 3:**
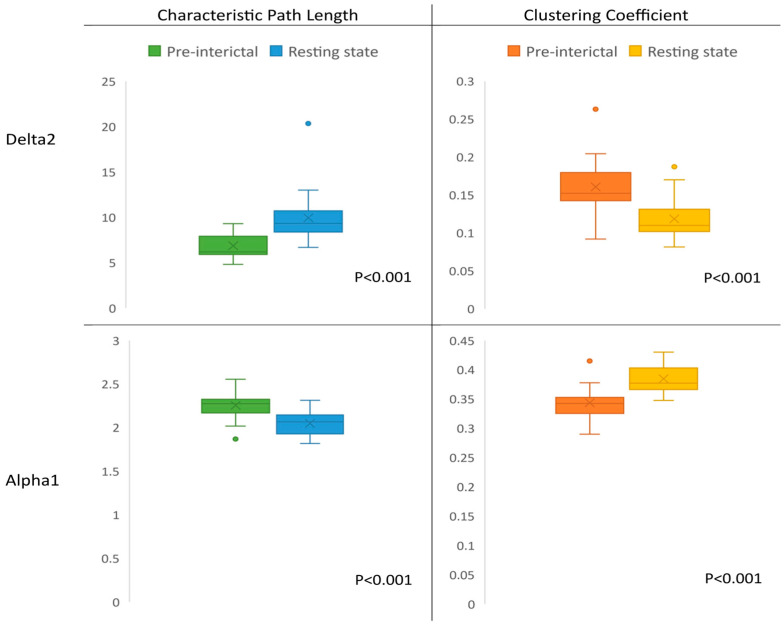
Network metrics in delta2 and alpha1 band during the pre-interictal and resting states.

During the pre-interictal state in the delta2 band, a significant network with increased connectivity was detected (T = 1.5, permutations = 5000, unadjusted *p* = 0.007, corrected *p* = 0.045). This network demonstrated a decreased characteristic path length (Mdn = 6.18) compared to the resting state (Mdn = 9.32), corrected *p* < 0.001, r = 0.98. It also displayed an increased clustering coefficient (Mdn = 0.15) in comparison with the resting state (Mdn = 0.11), corrected *p* < 0.001, r = −1.07. In the alpha1 band, a significant network with decreased connectivity was detected (T = 1.5, permutations = 5000, unadjusted *p* < 0.001, corrected *p* = 0.002), which displayed an increased characteristic path length (Mdn = 2.27) and a decreased clustering coefficient (Mdn = 0.34) compared to the resting state (Mdn characteristic path length = 2.06, Mdn clustering coefficient = 0.37), corrected *p* < 0.001, r = −0.97 and 1.07, respectively. These results are visualized in Figure 3.

### 3.3. Node Characteristics

In the delta2 band, the threshold for a significant hubness score was set to the median plus the interquartile range, since the distribution of the hubness values was not normal. The significant alterations of the spreading score emerged through the permutation analysis corrected via the FDR method. 

**Figure 4:**
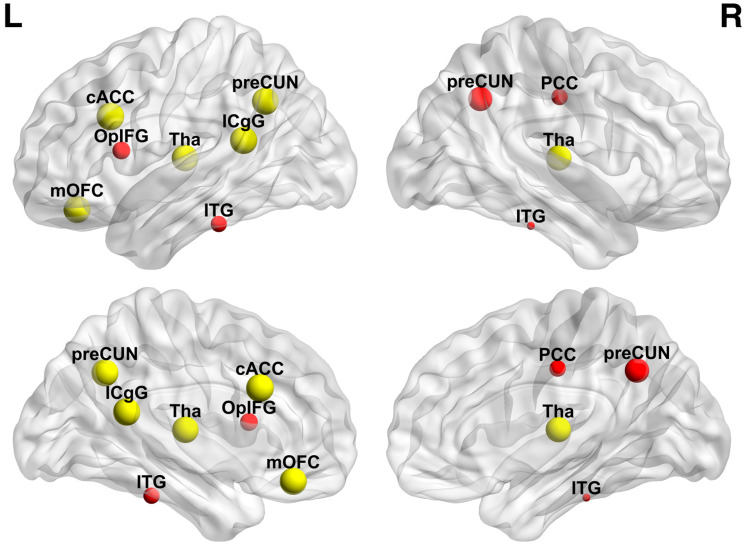
Hubs and spreader nodes in delta2 frequency band. Yellow nodes functioned as hubs. Red nodes demonstrated significantly altered spreading score. ITG: inferior temporal gyrus, ICgG: isthmus cingulate gyrus, OpIFG: pars opercularis, PCC: posterior cingulate, preCUN: precuneus, cAAC: caudal anterior cingulate cortex, Tha: thalamus, mOFC: medial orbitofrontal cortex.

The spreader nodes or hubs of the delta2 network were localized in the DMN, dorsal attention network (DAN), and the thalami. The left caudal anterior cingulate demonstrated the strongest hubness score, while the right precuneus exhibited the most significant increase in its spreading score (median difference = 13.366, permutation *p* value = 0.019, FRD corrected). The components of the network with significant hubness score and increment in the spreading score are visualized in Figure 4 and summarized in Table 2.

Respectively, in the alpha1 band, the distribution of hubness values was normal. A threshold, equal to the average plus one standard deviation, was correlated with the degree of loss of connectivity during the pre-interictal state (degree of desynchronization). Consequently, nodes with decreased connectivity were identified as desynchronized nodes.

Nodes in the alpha1 network, which were part of the DMN, DAN, and visual network (VIS), desynchronized at 8–10.5 Hz. The bilateral isthmus cingulate and the right lateral occipital gyrus showed the most significant loss of synchronization. No significant alterations were observed in the spreading score of nodes in alpha1 band. 

The results are summarized in Table 3. 

**Figure 5:**
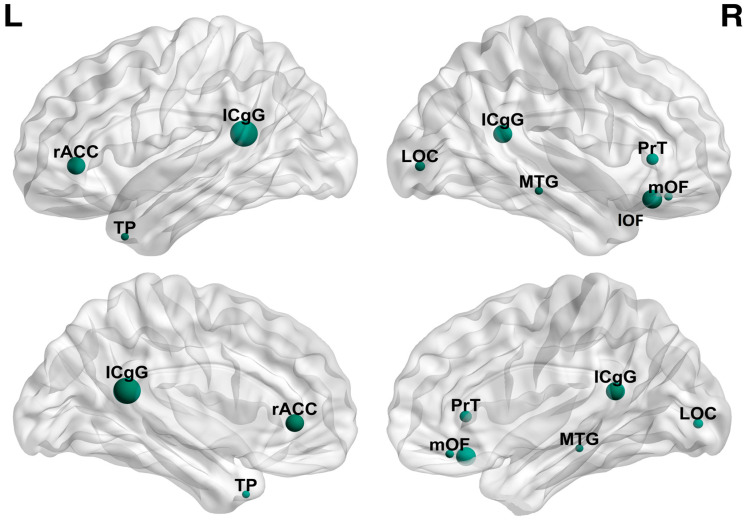
Desynchronized nodes in the alpha1 frequency band. ICgG: isthmus cingulate gyrus, LOC: lateral occipital gyrus, lOF: lateral orbitofrontal cortex, mOF: medial orbitofrontal cortex, MTG: middle temporal gyrus, PrT: pars triangularis, rACC: rostral anterior cingulate cortex.

The results of the degree of desynchronization in the alpha1 band are also visualized in Figure 5.

## 4. Discussion

In this HD-EEG study of a cohort of patients with IGEs, we aimed to elucidate the pre-interictal network state, preceding the interictal GSWDs. The connectivity analysis was conducted by estimating cortical lagged coherence during the pre-interictal and resting states. The network characteristics that we investigated were the characteristic path length and the mean clustering coefficient. Moreover, the nodal influence on the network was assessed through the estimation of hubness and spreading score.

We noticed that the pre-interictal state was characterized by the coupling of two networks with reversed connectivity attributes at 3–4 Hz (delta2 band) and 8–10.5 Hz (alpha1 band). The delta2 network exhibited an increased efficiency in information transfer and processing. On the contrary, the alpha1 one presented a decreased efficiency in these network features. Regarding the delta2 network, some nodes of the DMN and the DAN, as well as the thalami, exhibited an increase in either hubness or spreading score. We speculated that these nodes influenced the network towards an increased connectivity state in the delta2 band before the GSWDs. Simultaneously, in the alpha1 network, nodes of the DMN, DAN, and VIS displayed significant desynchronization. Interestingly, both the left isthmus cingulate and the right medial orbitofrontal participated in both networks.

The coupling of opposite dynamic networks may correspond to mechanisms leading to the formation of interictal GSWDs, while also inhibiting seizure activity. A recent MEG study on absence epilepsy compared preictal with pre-interictal activity. The authors showed that the interictal GSWDs were preceded by an increase in delta power and a decrease in alpha power [40]. Although the methodological approach of our study differs substantially, we provide evidence of network involvement in the same frequency bands during pre-interictal activity. We speculate, then, that the coupling of these network configurations preconditioned the generation of interictal GSWDs.

Our research results provided additional evidence highlighting the importance of the DMN in the pre-interictal state. Specifically, the right precuneus exhibited the greatest increase in spreading score, while the left caudal anterior cingulate demonstrated the highest hubness score. Previous studies have shown that the pathophysiology of GSWDs implicates the DMN and mainly the precuneus. Qin et al. discovered a pre-interictal stable network, which was predominantly located in the parietal and occipital regions in the alpha frequency band, with the precuneus operating as a highly connected hub node [41]. Moeller et al. documented activity changes in the precuneus several seconds before the onset of interictal GSWDs [42].

We also noted that the thalami demonstrated hub function in the delta2 network during the pre-interictal state, adding evidence that supports the contribution of the thalami to this state. A recent review on the role of the thalami in IGES suggested that there is a reciprocal increase in activity between the somatosensory cortex and the medial posterior thalamic nucleus. Moreover, the reticular thalamic nucleus reduces its inhibitory influence on the posterior thalamic nucleus, as well as on the ventrobasal complex. These interactions potentially facilitated the onset of spike–wave activity [43]. 

Additionally, our study revealed that the right lateral occipital gyrus, part of the VIS, exhibited significant desynchronization among other cortical areas in the alpha1 band. Recent studies emphasized the contribution of the visual network to the formation of GSWDs. A rodent model of genetic absences has revealed temporary desynchronization in cortical areas, such as the primary visual cortex, during spike–wave activity [44]. A clinical EEG/fMRI study on IGEs noted a decrease in synchrony of the occipital cortex during the GSWDs [45].

Moreover, we observed that the delta2 network manifested an increased clustering coefficient and a decreased characteristic path length during the pre-interictal state. According to functional connectivity studies on absence epilepsy during the preictal state, Jacobs-Brichford et al., utilizing sLORETA in EEG recordings, revealed a significant increase in power at 3 Hz [46]. Gupta et al., in an MEG study, identified a dynamic network with an increased clustering coefficient and a decreased characteristic path length during the preictal state. Researchers speculated that these network features allowed the emergence of GSWDs [47]. We hypothesized that the pre-interictal network configuration shares certain attributes with the preictal one, thereby possibly explaining the similarities observed in GSWD morphology and topology in the subsequent states.

The limitations of our study include the heterogeneity of the patient’s group regarding the IGE subsyndromes. Furthermore, all subjects were not drug-naïve. Antiseizure medications (ASMs) such as valproic acid have been reported to normalize functional connectivity in patients with IGEs [48]. The potential effects of ASMs were not analyzed due to the absence of an age-matched control group. Additionally, the lagged coherence metric, which was utilized for the connectivity analysis, estimated undirected linear dependencies, excluding the non-linear and directed ones, also evident in neural dynamics [35]. Finally, the rectification of the source maps before the difference estimation of sLORETA prevented the detection of differences between the equal values of opposite signs.

## 5. Conclusions

The analysis of the pre-interictal network state in IGEs, utilizing sLORETA and cortical lagged coherence, revealed two networks with opposite features. We demonstrated that this state was defined by the synchronization at 3–4 Hz, as well as the desynchronization at 8–10.5 Hz. In the 3–4 Hz network, we noted that the left caudal anterior cingulate had the highest hubness score, while the right precuneus had the most significant increment in the spreading score. In the 8–10.5 Hz network, we revealed the desynchronization mainly of the isthmus cingulate bilaterally, as well as the right lateral orbitofrontal cortex. The network analysis provided more evidence for the DMN, DAN, VIS, and thalami involvement in the pre-interictal state. This state might be considered as a favorable condition for the generation of interictal GSWDs. Further studies with a larger sample, as well as a control group, are required to validate our results.

## Figures and Tables

**Table 1:** Clinical and EEG characteristics.

Number of patients	21
Female/Male N (%)	10 (48%)/11 (52%)
Age in years (mean ± SD)	29.8 ± 12.4
Age at onset of epilepsy in years (mean ± SD)	17.1 ± 9.3
IGEs N (%)	
CAE	1 (5%)
JAE	6 (28.5%)
GTCSa	8 (38%)
JME	6 (28.5%)
ASMs	
Valproic	8 (43%)
Levetiracetam	4 (19%)
≥2 ASMs	9 (38%)
HD-EEG	
Number of channels Pre-interictal epochs (total/mean)	128 27/1.28

IGEs: idiopathic generalized epilepsy syndrome, CAE: childhood absence epilepsy, JAE: juvenile absence epilepsy, GTCSa: generalized tonic-clonic seizures alone, JME: juvenile myoclonic epilepsy, ASMs: anti-seizure medications, EEG: electroencephalogram, HD-EEG: high-density EEG.

**Table 2:** Functions of nodes at the delta2 band.

Function	Region of Interest	Hubness Score	Spreading Difference Score	RSN
Spreaders	Precuneus R	Subthr.	13.366 (*p* = 0.019)	DMN
	Posterior cingulate R	Subthr.	3.717 (*p* = 0.023)	DMN
	Inferior temporal L	Subthr.	2.439 (*p* = 0.022)	DAN
	Inferior temporal R	Subthr.	1.924 (*p* = 0.024)	DAN
	Pars opercularis L	Subthr.	1.912 (*p* = 0.045)	DAN
Hubs	Caudal anterior cingulate L	100	ns	DAN
	Isthmus cingulate L	95	ns	DMN
	Medial orbitofrontal L	92	ns	DMN
	Precuneus L	91	ns	DMN
	Thalamus L	85	ns	
	Thalamus R	74	ns	

Subthr.: subthreshold, ns: non-significant, DMN: default mode network, DAN: Dorsal Attention Network. Spreaders are nodes with statistically significant altered spreading score.

**Table 3:** Functions of nodes at the alpha1 band.

Function	Region of Interest	Dsync. Degree	RSN
Dsync. Nodes	Isthmus cingulate L	100	DAN
	Lateral orbitofrontal R	87	DMN
	Isthmus cingulate R	86	DAN
	Rostral anterior cingulate L	85	DAN
	Pars triangularis R	75	DAN
	Lateral occipital R	71	VIS
	Medial orbitofrontal R	69	DMN
	Middle temporal R	68	DAN

Dsync.: desynchronization, DMN: default mode network, DAN: Dorsal Attention Network, VIS: Visual Network.

## Data Availability

The data presented in this study are available on request from the corresponding author. The data are not publicly available due to Ethical restrictions.
